# Octahedral Tantalum Bromide Clusters as Catalysts for Light-Driven Hydrogen Evolution

**DOI:** 10.1021/acs.inorgchem.3c03045

**Published:** 2023-11-07

**Authors:** Jhon Sebastián Hernández, Daniela Guevara, Maxim Shamshurin, Enrico Benassi, Maxim N. Sokolov, Marta Feliz

**Affiliations:** † Instituto de Tecnología Química, Universitat Politècnica de València - Consejo Superior de Investigaciones Científicas (UPV-CSIC), Avd. de los Naranjos s/n, Valencia 46022, Spain; ‡ Nikolaev Institute of Inorganic Chemistry SB RAS, 3 Akad. Lavrentiev Ave., Novosibirsk 630090, Russian Federation; § Novosibirsk State University, 2 Pirogov Str., Novosibirsk 630090, Russian Federation

## Abstract

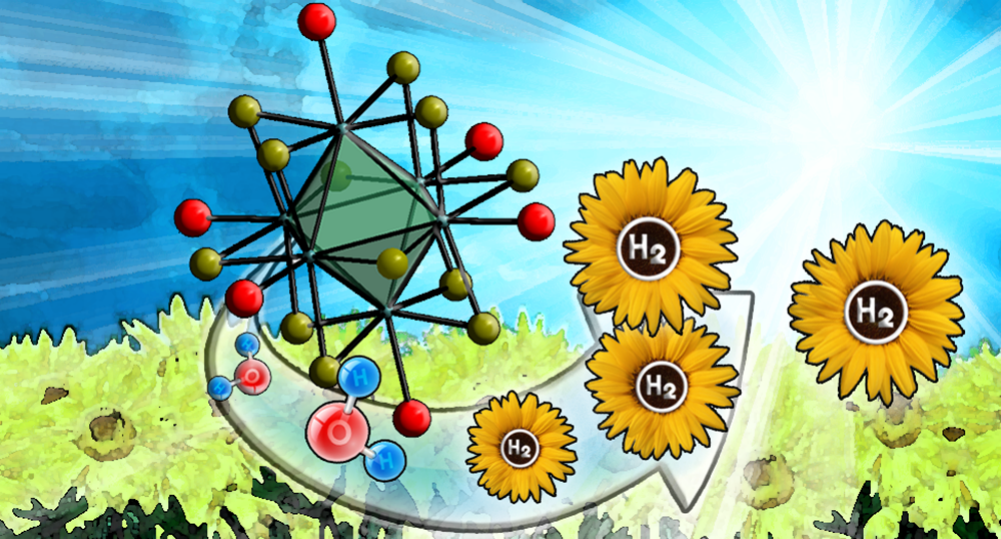

The development of an efficient hydrogen generation strategy from aqueous protons using sunlight is a current challenge aimed at the production of low-cost, easily accessible, renewable molecular hydrogen. For achieving this goal, non-noble metal containing and highly active catalysts for the hydrogen evolution reaction (HER) are desirable. Octahedral tantalum halide clusters {Ta_6_(μ-X)_12_}^2+^ (X = halogen) represent an emerging class of such HER photocatalysts. In this work, the photocatalytic properties of octahedral aqua tantalum bromide clusters toward HER and in acid and homogeneous aqueous conditions were investigated. The [{Ta_6_Br^i^_12_}Br^a^_2_(H_2_O)^a^_4_]·4H_2_O (i = inner ligand; a = apical ligand) compound is revealed to be an efficient precatalyst in acid (HBr) conditions and with methanol as the sacrificial agent. A response surface methodology (RSM) study was applied for the optimization of the HER conditions, considering the concentrations of both additives (methanol and HBr) as independent variables. An optimal H_2_ production of 11 mmol·g^–1^ (TON = 25) was achieved, which displays exceptional catalytic properties compared to regular Ta-based materials. The aqua tantalum bromide clusters assist in the photocatalytic hydrogen generation in agreement with energy-conversion schemes, and plausible active catalytic species and a reaction mechanism were proposed from computational and experimental perspectives.

## Abstract 2

Octahedral tantalum clusters are redox-active compounds. The [{Ta_6_Br^i^_12_}Br^a^_2_(H_2_O)^a^_4_]·4H_2_O (i = inner; a = apical ligands) cluster results in efficient precatalyst for the light-driven H_2_ production from aqueous protons in homogeneous conditions. The most active cluster species are *in situ* generated by apical ligand exchange reaction from aqueous media.

## Introduction

In the past few years, a renewed interest for group 5 and 6 octahedral halide-bridged clusters has revealed many outstanding properties of this family of compounds with prospects of applications in energy conversion, catalysis, radiology, and materials science.^1^ These entities belong to the large family of metal atom clusters,^2^ with {M_6_(μ_3_-X)_8_} (M = Mo, W) and {M_6_(μ_2_-X)_12_} (M = Nb, Ta; X = halogens) cluster units as robust central entities, coordinated by six labile terminal (apical) ligands. In particular, the octahedral tantalum clusters can be expressed by the general formula [{Ta_6_X^i^_12_}L^a^_6_]^*n*^, in which the six metal atoms are interconnected by direct metal–metal bonds and linked by 12 inner cluster ligands (X^i^, at the edge-bridging positions) and 6 apical ligands (L^a^, at the terminal positions).^3−6^ Studies on the catalytic properties the octahedral halide-bridged clusters and their cluster hybrids derivatives have mostly been focused on the molybdenum and niobium clusters as heterogeneous catalysts at elevated temperatures.^7^ In the past few years, these clusters have attracted interest in the field of the conversion of solar energy into clean fuels thanks to their optical and redox properties.^8,9^ In fact, octahedral metal-cluster-based hybrid systems combine the advantages of both molecular catalysts and semiconductors, viz., high catalytic activity, broad visible-light absorption, and long-term stability of light harvesters. Molecular clusters based on {Mo_6_(μ_3_-X_8_)}^4+^ (X = Br, I) cluster cores have been shown to be efficient catalysts in photoassisted water reduction.^3,10,11^ Their facile ligand and counterion exchange facilitates cluster immobilization onto graphene surfaces to build suitable nanohybrid nanostructured materials for H_2_ production. Recently, the coordinative anchoring of octahedral clusters onto graphene oxide (GO) has been extended to {Ta_6_Br^i^_12_} clusters, and the GO-cluster nanohybrids have demonstrated to be efficient photocatalysts in HER.^12^ Even though heterogeneous systems are expected to exhibit higher H_2_ production rates and longer stability than homogeneous ones, the activity of the nonsupported {Ta_6_Br^i^_12_}^2+^ clusters as photocatalytically reactive sites in HER and in homogeneous conditions remains unexplored, whereas Vogler and Kunkely reported noncatalytic photoredox reactivity toward water reduction.^13^ The study of these potential molecular cluster photocatalysts shows peculiar advantages because they are more exposed to reaction transformations and their chemical and photochemical properties may be tuned at the molecular level. However, the cluster stability can be affected upon irradiation and lead to decomposition into metal-based inorganic materials, which could play a catalytic role.^3,10,11,14^

In this work, the reactivity and photocatalytic activity of the aqueous {Ta_6_Br^i^_12_}^2+^ cluster species toward the HER from protons and light were studied under homogeneous conditions. Cluster stability, recyclability, and reaction kinetics along with a plausible reaction mechanism proposed on the basis of computational and experimental data will be discussed.

## Experimental Section

### Materials and Methods

Hydrobromic acid (ACS Reagent 48%), methanol (ACS Reagent ≥99.8%), acetic acid (ACS Reagent 99.9%), l-ascorbic acid (ACS Reagent ≥99%), and lactic acid (ACS Reagent 98%) were obtained for commercial resources (Sigma-Aldrich). The ultrapure water was produced with the Milli-Q eq 7000 Type 1 water purification system. No uncommon hazards are noted. The K_4_[{Ta_6_Br^i^_12_}Br^a^_6_] and the [{Ta_6_Br^i^_12_}Br^a^_2_(H_2_O)^a^_4_]·4H_2_O solid materials were prepared in high purity (99%) and by our optimized Koknat’s procedure.^12,15^ The [{Ta_6_Br^i^_12_}Br^a^_2_(H_2_O)^a^_4_]·4H_2_O represents detailed structure of the previously reported {Ta_6_Br^i^_14_}·8H_2_O compound^15^ and will be used throughout the text. Once prepared, solid [{Ta_6_Br^i^_12_}Br^a^_2_(H_2_O)^a^_4_]·4H_2_O was stored in a desiccator, since it is known that the number of solvation molecules varies depending on the variations in the environmental conditions.^16^

Freshly prepared sample of [{Ta_6_Br^i^_12_}Br^a^_2_(H_2_O)^a^_4_]·4H_2_O was dissolved in water at room temperature under continuous stirring to yield an intense green solution mainly associated with the aquated [{Ta_6_Br^i^_12_}(H_2_O)_6_]^2+^ cluster species,^12^ according to the established protocols.^15,17^ The identification of the resulting aqua cluster species was achieved by UV–vis and photoluminescence experiments in an aqueous solution. The purity was determined (99%) by applying the Lambert–Beer equation, assuming the molar extinction coefficient (ε (640 nm) = 6600 L·mol^–1^·cm^–1^) at 640 nm, as reported for the hydrated {Ta_6_Br^i^_12_}^2+^ cluster complex.^13^

### Instrumentation

UV–vis absorption analyses were performed using a Varian Cary 50 UV–vis Agilent analyzer equipped with a Xe lamp as a light source and a Czerny–Turner model dual beam monochromator and with 10 × 10 mm quartz cuvettes. Cuvettes were equipped with screw caps with silicon septum were used for all spectrophotometric measurements done under an Ar atmosphere. For samples comprising aqueous cluster solutions in the presence of other additives, the spectra were registered with a dilution factor of 5. Steady-state photoluminescence (PL) measurements were recorded in an Edinburgh Instruments FLS1100 spectrofluorometer using a 450 W xenon lamp light equipped with a double monochromator for excitation and emission coupled to a visible photomultiplier or an InGaAs detector. Mass spectrometry (MS) spectra were performed on a Xevo QTof instrument using electrospray ionization technique (ESI). The cone voltage was set between 5 and 30 V to identify the most stable species. The composition of the peaks with the highest mass-to-charge ratio (*m*/*z*) was as-signed by comparison of the experimental isotopic distribution with the theoretical one, which is obtained using the MassLynx NT software package. Molecular hydrogen production was monitored by gas chromatography (GC) on the Agilent 490 Micro GC System, equipped with a column coated with a zeolite molecular sieve (CP-Molsieve 5 Å, Agilent J&W) and a conductivity detector (TCD). Ar was taken as the carrier gas, and the flow rate was set to 5 mL·min^–1^. The inlet and detector temperatures in the GC run were 110 and 220 °C, respectively, and the isothermal oven temperature profile was set at 62 °C with an initial column pressure of 15 psi. The pH analyses were performed with a Phoenix EC-40 pH instrument. Cyclic voltammograms (CV) were recorded by using a 797 VA Computrace system (Metrohm, Switzerland). All measurements were performed with a conventional three-electrode configuration consisting of a glassy carbon working and a platinum auxiliary electrode and an Ag/AgCl/KCl reference electrode. The solvent was H_2_O. KNO_3_ (1 M) was used as a supporting electrolyte. The concentration of the cluster was less than 1mΜ. Redox potential values (*E*_1/2_) were determined as (*E*_*a*_ + *E*_*c*_)/2, where *E*_*a*_ and *E*_*c*_ are anodic and cathodic peak potentials, respectively.

### Photochemical H_2_ Evolution Experiments

All the manipulations were performed under an argon atmosphere and using Schlenk techniques. For the photoreactivity and photocatalytic reactions, the aqueous mixtures were previously deoxygenated by bubbling dry argon for at least half an hour.

The photoreactivity experiments performed for *in situ/in operando* UV–vis monitoring were carried out in an UV–vis quartz cuvette and under an argon atmosphere. A Hamamatsu Xe lamp was used as the radiation source with a spot light positioned perpendicularly at 1.5 cm from the cuvette wall. Measurements were monitored every 5 min during the first hour of reaction and then every 30 min.

The HER experiments directed to molecular hydrogen quantification were carried out under a rigorous inert atmosphere and according to the following general procedure: the chosen reactor was a cylindrical quartz reactor of 55 mL and 144 mm in diameter, equipped with a valve coupled to a manometer to determine the pressure into the reactor. Figure S1 illustrates the experimental layout for the experiments. For the optimization tests, 7.7 μmol of cluster (18 mg of [{Ta_6_Br^i^_12_}Br^a^_2_(H_2_O)^a^_4_]·4H_2_O was dissolved in 15 mL of pure water or the chosen aqueous mixture to obtain solutions with a concentration of 5 × 10^–4^ M. For optimizing the catalytic properties, 10, 5, and 1 mg of the cluster compound were employed. The system was then purged with argon bubbling, and it was pressurized to a pressure between 0.25–0.3 bar. In order to ensure that the cluster fully dissolved, the reactor was subjected to ultrasound for 3 min. The reactor temperature was set at 25 °C by means of a cooling system, and the homogeneity of the solution remained stable with constant magnetic stirring. Next, the vessel was irradiated with a Hamamatsu Xe lamp with a spot light placed at a distance of 5 cm above the reactor surface. The gas phase samples (500 μL) were collected with a Hamilton syringe and injected to the GC-TCD spectrometer. The hydrogen peak area was calculated to the corresponding concentration using the standard calibration curve as reference. The micromoles of hydrogen produced were calculated taking into account the ideal gas law (*n* = *PV*/*RT*) and these quantifications were determined considering the [{Ta_6_Br^i^_12_}Br^a^_2_(H_2_O)^a^_4_]·4H_2_O as a photocatalyst. Control experiments showed the detection of atmospheric gases, exclusively. The absorption spectra and pH of the catalytic solution were analyzed before and after reaction. MS-ESI was employed for the identification of plausible cluster species during the HER reaction in methanol/water mixtures.

In order to evaluate the materials’ stability, reuse tests were carried out for four cycles under the same conditions as the initial experiments. The cluster material was recovered by precipitation with HBr and, after careful decantation, the resulting green solid was isolated and dried at 50 °C overnight. The H_2_ produced is reported as percentage with respect to the value of hydrogen obtained in the first use.

### Experimental Design and Data Analysis

The Design RStudio Software was used for the statistical design of experiments and data analysis.^18^ In this study, the Central Composite Design (CCD) and Response Surface Methodologies (RSM)^19−22^ were applied in order to optimize the two variables, methanol and bromidic acid concentrations (mol·L^–1^), to obtain the highest hydrogen production (μmol·g^–1^) of the system reaction. The CCD consists in an experimental design, used in RSM in order to obtain a second order (quadratic) model for one response variable, namely, the hydrogen produced in a photochemical reactions under specific conditions, without the need to use a full three level factorial design.^21,22^ The design proposed in this research corresponds to a 3^2^ full factorial design that involves four experiments (experiment numbers from 10 to 13) as replicates of the central point. With the aim to determine the range of HBr concentration, the acid from 0.1 mol·L^–1^ was added until precipitation at 2.0 mol·L^–1^. As a result of this study, we chose 1.0 mol·L^–1^ as the central concentration point for HBr. In the case of methanol, the central point was set to 4.94 mol·L^–1^. To prevent the effect of the selection bias, all the experiments were doing randomly.

In order to obtain the optimum concentration of HBr and MeOH (independent parameters), the hydrogen produced in the process was analyzed as a response variable. The quadratic equation model for the estimation of the optimal conditions was obtained according to the eq (1):
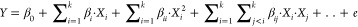
1where β_0_ indicates the offset (intercept), β_*i*_ is the linear coefficients, β_*ii*_ is the pure quadratic coefficients, β_*ij*_ the spurious quadratic coefficients, *k* the number of factors studied, and *e* the random error.

In the study, the analysis of variance (ANOVA) was used for the graphical analyses of the data in order to obtain the interactions between the response and the independents variables. The dimensional plot and its respective contour plot were obtained using the same program for the data treatment (R and Rstudio) and based on the effects of the two independent variables.^18^

### Computational Details

The molecular geometries of the electronic ground state of tantalum bromide clusters [{Ta_6_Br_12_}(H_2_O)_6_]^*n*^, [{Ta_6_Br_12_}(H_2_O)_5_(OH)]^*n–*1^ and [{Ta_6_Br_12_}(H_2_O)_5_(MeOH)]^*n*^ (*n* = 2, 3, 4) were fully optimized both in gas and solvated phase at the density functional theory (DFT) level using the hybrid exchange correlation functional B3LYP,^23−25^ coupled with the triple−ζ Ahlrichs’ Def2–TZVPP basis.^26,27^ The geometries and energies included in this work refer to calculations in the solvated phase. No empirical dispersion was included. The choice of functional and basis set was based on a previous investigation on similar compounds.^28^ The optimized geometries were subsequently submitted to frequency calculation (in harmonic approximation). No negative frequencies were found. Thermochemical quantities (at *T* = 298.15 K and *p* = 1 atm) were computed at the same level of theory. The atomic charges were computed by means of two different approaches, *viz*., by Mulliken’s population analysis and the atomic polar tensor (APT) derived charges.^29^

The excitation energy and oscillator strength first ten singlet and ten triplet vertically excited electronic states were computed at TD-DFT CAM-B3LYP Def2–TZVPP level;^30^ the states’ nature was contextually characterized.

The solvent (water) was treated implicitly, within the framework of the polarizable continuum model using the integral equation formalism variant (IEF-PCM).^31^ The values of static dielectric constant (ε) and refraction index (*n*^2^) were taken from the literature.^32^ The cavitation radii were the standard UFF radii, scaled by a factor α = 1.1; the scale factor for the metal atoms was modified to check the consistency of the results. Both equilibrium (*eq*) and nonequilibrium (*neq*) regimes as well as for the excited electronic states linear response (LR) and state specific (SS) solvation approaches were employed.

Ground state geometry optimizations and frequency calculations were repeated at the DFT M06-2X/Def2-TZVPP level, including solvents' effects by means of the SMD model as this level is acknowledged as more accurate for energy predictions.^33^

For all the calculations, the integration grid for the electronic density was set to 250 radial shells and 974 angular points. Accuracy for the two–electron integrals and their derivatives was set at 10^–14^ a.u. The self-consistent field (SCF) algorithm used was the quadratically convergent procedure designed by Bacskay,^34^ a method which is acknowledged as slower but more reliable than regular SCF with DIIS extrapolation. The convergence criteria for SCF were set at 10^–12^ for root-mean-square (RMS) change in the density matrix and at 10^–10^ for maximum change in the density matrix. Convergence criteria for geometry optimizations were set at 2 × 10^–6^ a.u. for maximum force, 1 × 10^–6^ au for RMS force, 6 × 10^–6^ au for maximum displacement, and 4 × 10^–6^ au for RMS displacement. All calculations were performed using the GAUSSIAN G16.C01 package.^35^

## Results and Discussion

### Study of [{Ta_6_Br^i^_12_}Br^a^_2_(H_2_O)^a^_4_]·4H_2_O in Photochemical HER

In order to assess the catalytic activity of the [{Ta_6_Br^i^_12_}Br^a^_2_(H_2_O)^a^_4_]·4H_2_O cluster with water, photoreactivity experiments were first conducted in strictly homogeneous and inert conditions. Dissolution of [{Ta_6_Br^i^_12_}Br^a^_2_(H_2_O)^a^_4_]·4H_2_O provided aqueous cluster species, mainly [{Ta_6_Br^i^_12_}(H_2_O)^a^_6_]^2+^, and the resulting deep green solution was identified by UV–vis spectroscopy (Figure S2). Vogler and Kunkely established that the proton reduction reaction is achieved by simultaneous oxidation of the complex, promoted upon light irradiation and in the presence of acid as a proton source (eq 2).^13,17^ Hydrobromic acid was chosen in order to prevent eventual halogen exchange within the {Ta_6_Br^i^_12_} cluster core under the experimental conditions.
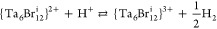
2

**Figure 1:**
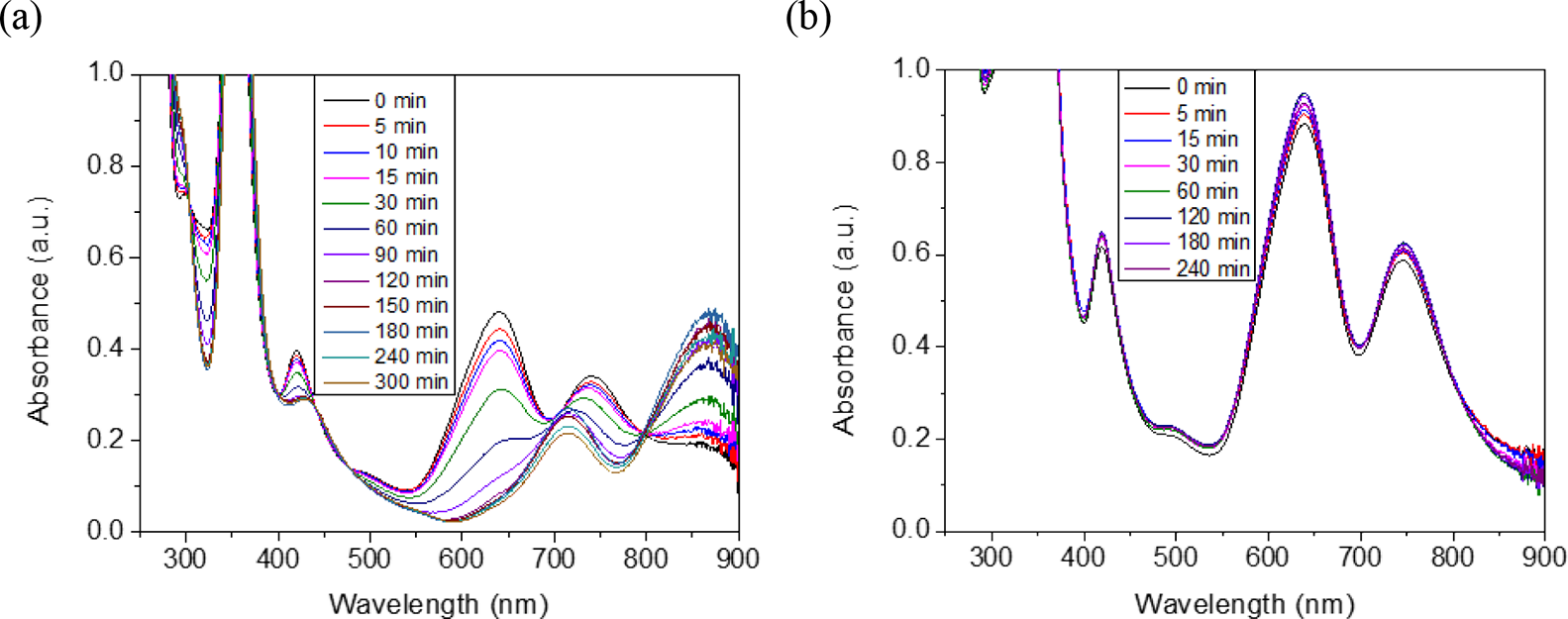
UV–vis spectra after photolysis of [{Ta_6_Br^i^_12_}Br^a^_2_(H_2_O)^a^_4_]·4H_2_O in the presence (a) and absence (b) of HBr 1.0 mol·L^–1^ (cluster concentration: 1.2 × 10^–4^ mol·L^–1^).

Two irradiation experiments were conducted, with and without HBr of 1.0 mol·L^–1^ in aqueous solution, respectively. The progress of the UV–vis spectra was monitored *in situ/in operando* in a spectrometric cuvette, and the H_2_ generated was quantified when the reaction took place in a photochemical reactor. The UV–vis monitoring showed evolution of the characteristic spectra of [{Ta_6_Br^i^_12_}(H_2_O)^a^_6_]^2+^ in acidic conditions during a 5 h irradiation, with significant changes of the bands between 400 and 900 nm (Figure 1a), similar to that reported in HCl solution.^13^ The new bands are associated with the *in situ* generation of 3+ oxidized cluster species. In the absence of the acid, the characteristic bands of [{Ta_6_Br^i^_12_}(H_2_O)^a^_6_]^2+^ persisted upon a 4 h irradiation (Figure 1b). This highlights the fact that no photoredox reaction takes place in the absence of the acid. However, a progressive and slight decrease of the intensity of these bands with reaction time was noticed, which was associated with a low-yield decomposition or hydrolysis of the complex toward Ta_2_O_5_ under experimental conditions.

GC results (Figure S3) confirmed the production of H_2_ according to 2. A small amount of H_2_ (3 μmol·gr^–1^ of [{Ta_6_Br^i^_12_}Br^a^_2_(H_2_O)^a^_4_]·4H_2_O cluster) was obtained after 1 h of irradiation in HBr, and this production remained stable for at least 6 h (Figure S3), confirming that no hydrolysis of the cluster is achieved in acidic conditions. This low hydrogen amount is comparable to that described in the presence of HCl, even though in that case, the features of the lamp used and the reaction time were not specified,^13^ and corresponds to a 1% cluster conversion to the oxidized {Ta_6_Br^i^_12_}^3+^ cluster species, according to 2. The progressive increase of the new cluster absorption bands (Figure 1(a)), associated with the 3+ oxidized cluster species, is also related to the light-promoted generation of {Ta_6_Br^i^_12_}^4+^ cluster core species. The associated absorption bands are not detected, whereas the generation of 3+ species is promoted by the comproportionation reaction involving 2+ and 4+ species, as depicted in 3.^13^ We performed a cyclic voltammetry experiment in order to verify the reversibility of the oxidized species, and two consecutive and quasi-reversible one-electron transfer processes are detected (Figure S4), associated with a consecutive two-step oxidation of the aqueous {Ta_6_Br^i^_12_}^2+^ to lead {Ta_6_Br^i^_12_}^3+^ and {Ta_6_Br^i^_12_}^4+^ species. The first oxidation potential appears at 0.378 V (Δ*E* = 66 mV), and the second at 0.669 V (Δ*E* = 65 mV), which are similar to the previously published data recorded in acidic conditions.^36^ The positioning of the frontier orbitals from the redox and NIR absorption properties of the 2+ and 3+ cluster species suggests that the proton to H_2_ photoreduction is thermodynamically favorable for both species. In fact, the two-electron transfer from {Ta_6_Br^i^_12_}^2+^ species to aqueous protons is energetically more favorable to produce H_2_ and {Ta_6_Br^i^_12_}^4+^ in a 1:1 stoichiometric ratio, in agreement to studies found in the literature.^13^ No HER was recorded in pure water (Figure S3), which confirms the need of protons for promoting the photoredox reaction, nor from an aqueous solution of the cluster (without acid), suggesting that the presence of small amounts of Ta_2_O_5_ does not promote the H_2_ production under light.
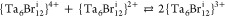
3

**Figure 2:**
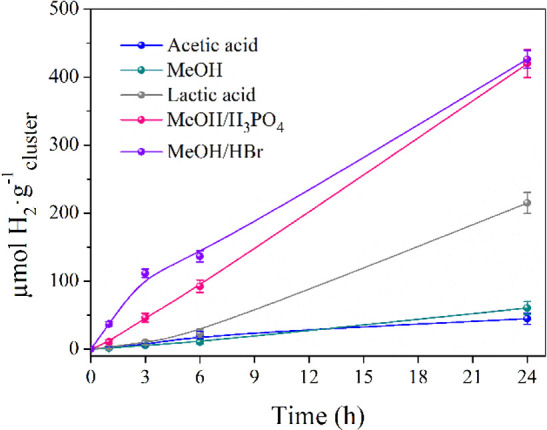
HER screening of [{Ta_6_Br^i^_12_}Br^a^_2_(H_2_O)^a^_4_]·4H_2_O in the presence of a sacrificial electron donor (*viz.*, acetic acid (3.50 mol·L^–1^), lactic acid (2.66 mol·L^–1^), and methanol (4.94 mol·L^–1^)) and acids (*viz.*, HBr (1.0 mol·L^–1^) and H_3_PO_4_ (3.84 mol·L^–1^)).

In a second stage, the hydrogen production promoted by [{Ta_6_Br^i^_12_}Br^a^_2_(H_2_O)^a^_4_]·4H_2_O was optimized in the presence of sacrificial electron donors and was monitored by GC and absorption spectroscopy. There is a wide scope of additional sacrificial reagents that are also commonly used in H_2_ generation from protons.^37,38^ Among them, we chose cheap and abundant alcohol, such as methanol, and acid representatives, as acetic, lactic, and ascorbic acids, which also play the role as proton donors, thus avoiding the use of HBr. Longer irradiation times (24 h) were achieved, and sacrificial additives were used in 20% v/v. Whereas the photochemical reaction in the presence of ascorbic acid did not produce H_2_, upon 3 h of irradiation, the amount of H_2_ produced by the acetic and lactic acids (22 μmol·g^–1^) was independent of the acid used (Figure 2) and above the amount registered in the absence of any sacrificial electron donor agent. The yield of H_2_ obtained rose considerably on going from acetic acid (45 μmol·g^–1^) to lactic acid (215 μmol·g^–1^) at the end of the reaction (Figure 2), which indicates that the most efficient reaction corresponds to the lowest p*K*_a_ of lactic acid. At this point, the absorption spectra showed that the cluster remained practically stable throughout the runs and revealed the presence of cluster species only in its reduced {Ta_6_Br^i^_12_}^2+^ form, as expected for an ideal catalytic cycle (Figure S5a,b). Cluster hydrolysis was prevented by the use of lactic acid, and the pH remained unaltered during the reaction (pH = 0.68), whereas a slight cluster decomposition detected in the presence of acetic acid may be ascribed to the less acidic conditions (pH 1.27 and 1.29 before and after the reaction, respectively).

Methanol was proposed as an alternative sacrificial. Its use is attractive because of the well-known electron donating ability and its easy removal (thanks to its high volatility). In addition, the bonding of alcohols in the apical positions of the {Ta_6_X^i^_12_}^2+^ (X^i^ = Cl, Br) cluster cores may play a role in the formation of cluster intermediates along the reaction.^39−41^ Due to the low p*K*_a_ of methanol, it was also employed in combination with strong acids, such as HBr and H_3_PO_4_. Using methanol/H_3_PO_4_ and MeOH/HBr mixtures, higher H_2_ amounts (420 and 426 μmol·g^–1^, respectively) were obtained after 24 h, and the fastest production rate was recorded in the presence of HBr during the first 3 h of irradiation (Figure 2). These H_2_ yields are 2-fold higher than that obtained with lactic acid. As a control test, the activity recorded with methanol as the sole additive dropped to 61 μmol·g^–1^ (Figure 2). The UV–vis spectra (Figure S5c,d) established that the cluster species decomposed slightly in pure methanol, whereas they remained intact in the presence of the methanol/HBr mixture. The need for the acid conditions to preserve the cluster stability and promote hydrogen generation and is again confirmed. Surprisingly, the evolution of the reaction mixture with phosphoric acid toward oxidation and cluster decomposition was detected by UV–vis (Figure S5e) after exposure to air.

The use of MeOH/HBr mixtures was proposed as indoneous in order to achieve the largest hydrogen production and to preserve tantalum cluster species. Control experiments confirmed the need for light, the cluster, and the additives to achieve these results (Table S1). In order to optimize the performance of the Ta-cluster/MeOH/HBr system, an appropriate experimental study was designed following response surface methodology (RSM) via Central Compose Design (CCD). HBr and MeOH concentrations were chosen as independent variables. The CCD matrix was obtained with 13 randomized experiments, and the results and predicted values are presented in Table 1. The maximum hydrogen production achieved was about 428 μmol·g^–1^ for the conditions defined in the experimental design, with a percentage error less than 5% in most cases. It is worth noting that the percentage error in the calculated values changes for HBr concentrations higher than 1.5 M due to the precipitation of the cluster and the modification of the reaction system since it is no longer a homogeneous reaction, as confirmed by the absorption spectra recorded under these conditions (Figure S6a). The increase in error was also attributed to the highest MeOH concentrations (≥7.41 mol·L^–1^). Absorption spectra showed that the cluster evolved toward oxidation and cluster decomposition in the presence of excess of alcohol in alcohol/acid mixtures (Figure S6b,c), which thus prevents its role as the active species.

**Table 1:** CCD, Predictive Values, and Experimental Results.^a^

**exp no**	**MeOH**(mol·L^–1^)	**HBr**(mol·L^–1^)	**calculated H**_**2**_(μmol·g^–1^)	**experimental H**_**2**_(μmol·g^–1^)	**error (%)**
1	2.47 (−1)	0.5 (−1)	346.11	337	2.49
2	7.41 (1)	0.5 (−1)	340.60	324	4.92
3	2.47 (−1)	1.5 (1)	177.72	174	2.21
4	7.41 (1)	1.5 (1)	172.21	100	41.7
5	1.24 (−0.5)	1.0 (0)	205.64	212	3.20
6	8.65 (0.5)	1.0 (0)	196.12	221	12.7
7	4.94 (0)	0.1 (−0.5)	354.59	371	4.69
8	4.94 (0)	1.9 (0.5)	51.51	66	29.1
9	4.94 (0)	1.0 (0)	434.20	416	4.1
10	4.94 (0)	1.0 (0)	434.20	414	4.56
11	4.94 (0)	1.0 (0)	434.20	410	5.59
12	4.94 (0)	1.0 (0)	434.20	428	1.34
13	4.94 (0)	1.0 (0)	434.20	411	5.26

a The coded values (reported in brackets) for HBr (*A*) and methanol (*B*) concentration were set at five levels: – 1 (minimum), – 0.5 (minimal star point), 0 (central), + 0.5 (maximal star point), and +1 (maximum).

**Figure 3:**
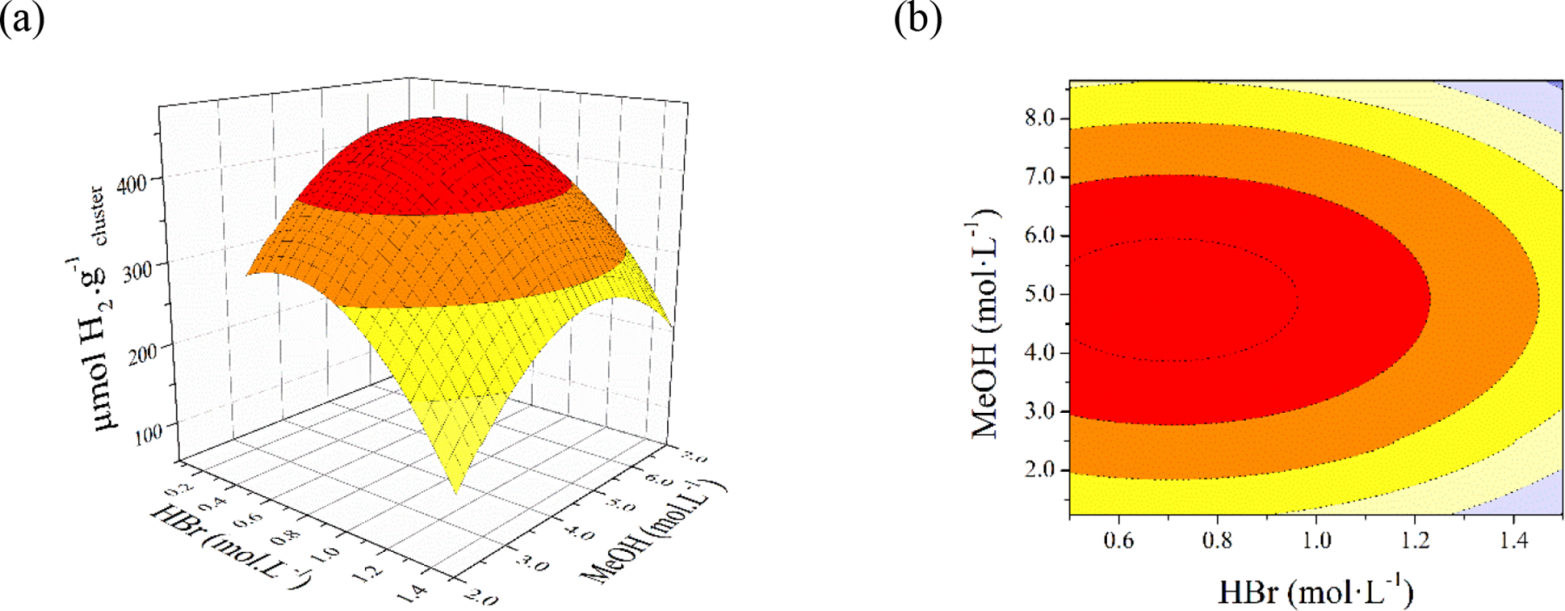
Surface (a) and contour (b) graphs representing the effect of MeOH and HBr concentrations on the hydrogen production.

Based on the data analysis (Table S1), an empirical quadratic equation was proposed for the H_2_ production using the tantalum bromide cluster as the active species (eq. 4).
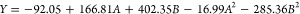
4where *Y* indicates the H_2_ amount (μmol·g^–1^), *A* is the methanol concentration (v/v), and *B* is the HBr concentration (mol·L^–1^). The ANOVA analysis of the data (Table S1), calculated *p* value (*p* = 0.00005 < 0.005) and the lack of a fit value (0.003 < 0.05) led us to confirm that the proposed equation matches with the experimental data. The effect of variables *A* and *B* on the hydrogen production (*Y*) is illustrated in Figure 3, which shows that the maximum value for *Y* is achieved when the MeOH and HBr concentrations are 4.83 and 0.7 mol·L^–1^, respectively (optimal conditions). In addition, the effect of the acid concentration on variable *Y* is more marked due to cluster precipitation caused by an excess of HBr.

**Figure 4:**
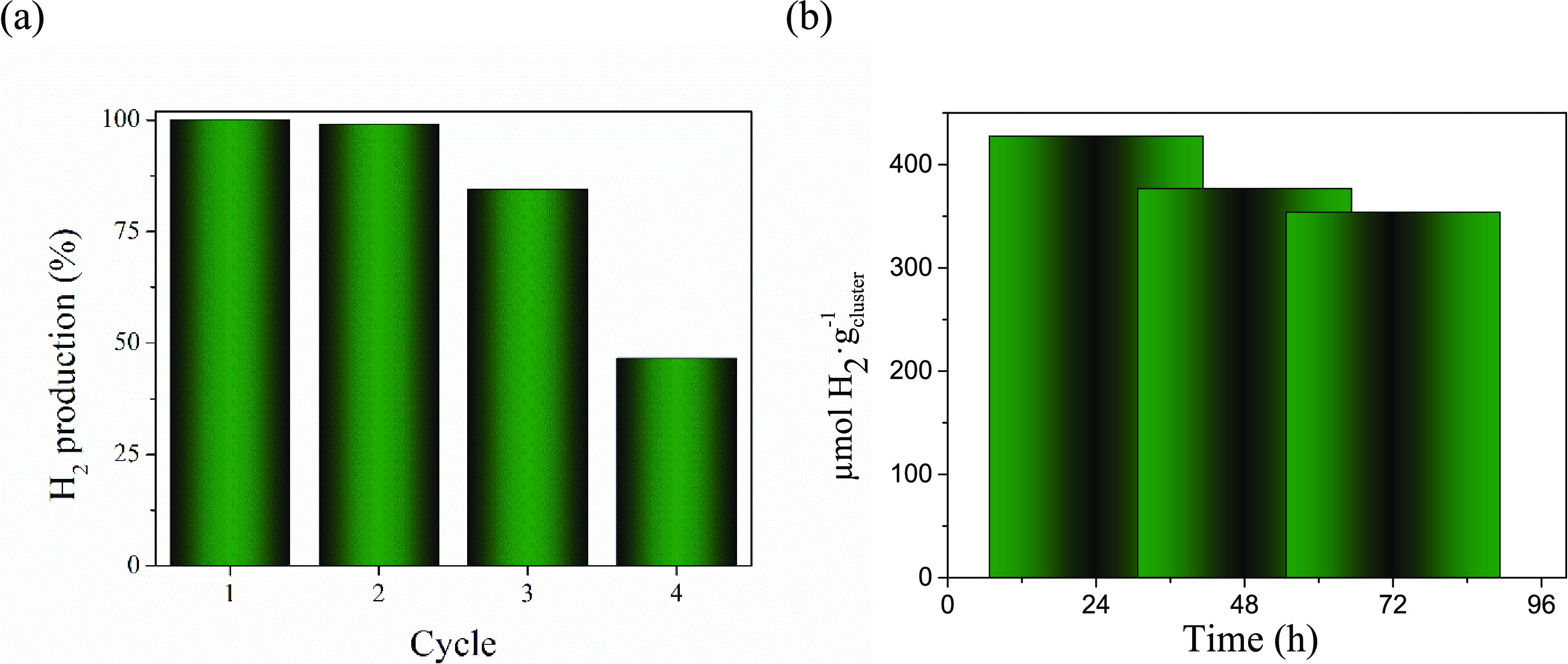
(a) Recycling of [{Ta_6_Br^i^_12_}Br^a^_2_(H_2_O)^a^_4_]·4H_2_O under optimal reaction conditions; (b) representation of the H_2_ evolution from the reaction mixture exposed to long irradiation time.

The optimal conditions were applied for an additional experiment, and the hydrogen quantity obtained was 442 μmol·g^–1^, which is within the experimental error (3.6%). The tantalum cluster-based material was recovered as a green solid, and four recycling experiments were carried out following the optimal conditions described above. The cluster was stable after two reuses, but its performance in terms of activity halved after the third reuse (Figure 4a), probably due to the cluster decomposition associated with an aging of the recovered cluster after long exposure to irradiation. In an additional experiment, we monitored the HER activity during 3 days of illumination and observed that the activity decreases progressively under reaction conditions (Figure 4b). Then, the color of the reaction mixture progressively bleached, and a white precipitate appeared at the end of the reaction, associated with the formation of Ta_2_O_5_ by cluster hydrolysis. These observations support the loss of stability of the *in situ* generated tantalum cluster active species in the reaction media. Whereas Ta_2_O_5_ is a recognized as a photocatalyst, generally combined with noble metal cocatalysts,^42^ we can infer from recycling and long-irradiation experiments that the Ta_2_O_5_ produced does not contribute in the increase of the H_2_ production.

The efficiency of the activity of [{Ta_6_Br^i^_12_}Br^a^_2_(H_2_O)^a^_4_]·4H_2_O (18 μmol·g^–1^·h^–1^) remained constant even when less quantity of cluster compound is present (10 vs 18 mg), and in both cases, TON = 1 agrees with an stoichiometric transformation. The catalytic performance of the Ta_6_ material was assessed by decreasing the amount of the cluster inverted during the reaction. Thus, we repeated experiments with 5 and 1 mg of cluster loading and obtained productions of 1.1 (TON = 3) and 11.0 mmol·g^–1^ (TON = 25), respectively. The catalytic performance of the HER achieved in the presence of the minimum cluster amount corresponds to 442 μmol·g^–1^·h^–1^ (TOF = 3 × 10^–4^ s^–1^ per cluster molecule). This value was 126-fold higher than that reported in the photoreduction of water vapor using the derived hybrid material {Ta_6_Br^i^_12_}@GO^12^ and 1 order of magnitude higher than the activities reported for tantalum oxides, oxynitrides, and nitrides in heterogeneous conditions, such as MTaO_3_ (M = Li, Na, K, Mg), BaTa_2_O_6_,^43^ Ta_2_O_5_,^44^ TaON,^45^ and Ta_3_N_5_ nanoparticles.^46^ It is worth noting that the optimal catalytic performance of the Ta cluster improves the activities reported for tantalum solids in the presence of Pt as a cocatalyst and with more powered irradiation lamps. This is an additional advantageous feature from the point of view of sustainable chemistry, since the use of noble cocatalysts is avoided. The catalytic activity of the [{Ta_6_Br^i^_12_}Br^a^_2_(H_2_O)^a^_4_]·4H_2_O cluster is in the same order than that achieved in homogeneous conditions in the presence of octahedral {Mo_6_Br^i^_8_}^4+^ cluster core catalysts (641 μmol·g^–1^·h^–1^ for (Et_4_N)_2_[{Mo_6_Br^i^_8_}F^a^_6_]^10^), with the advantage that the [{Ta_6_Br^i^_12_}Br^a^_2_(H_2_O)^a^_4_]·4H_2_O material stands out for its robustness in solution, as shown by the UV–vis spectra registered after reaction (Figure S7) conditions and the recyclability experiments.

**Figure 5:**
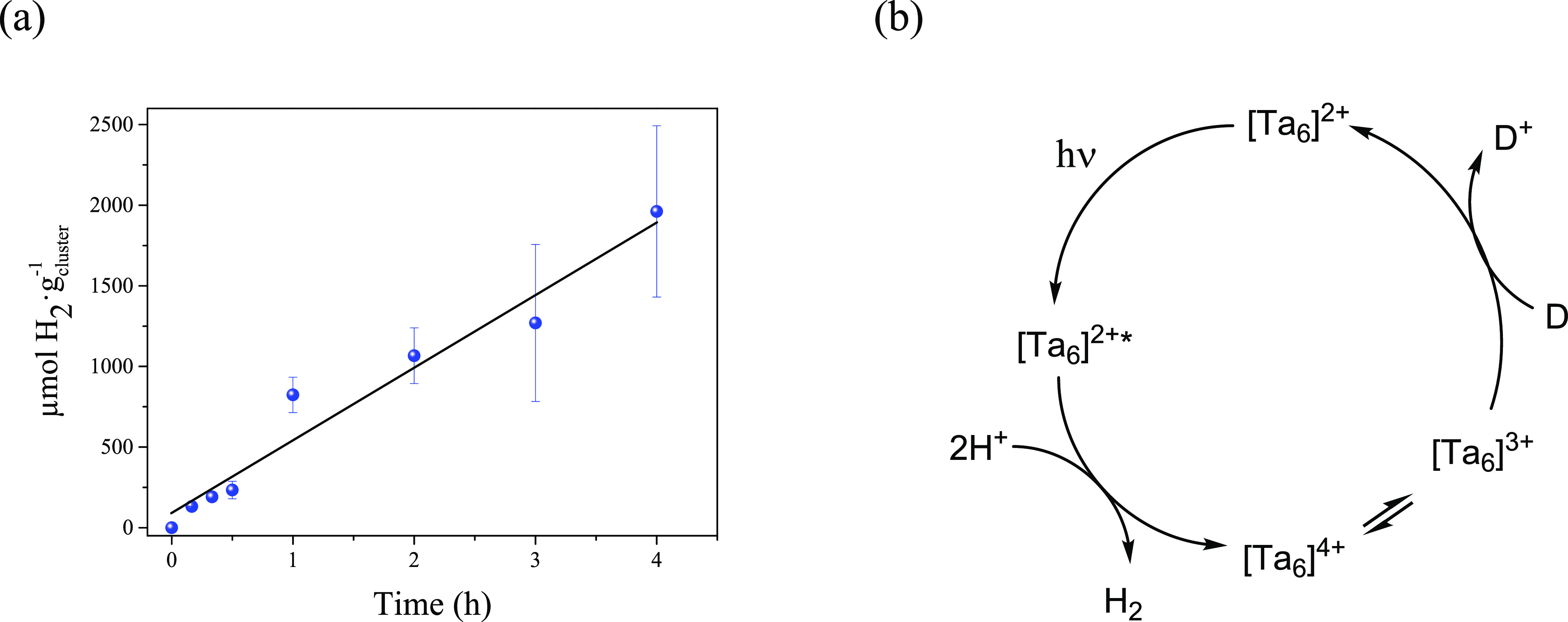
(a) Kinetic study and reaction rates of the UV–vis light driven H_2_ generation catalyzed by [(Ta_6_Br^i^_12_}Br^a^_2_(H_2_O)^a^_4_]·4H_2_O (1 mg) under optimal conditions; (b) proposed oxidative quenching mechanism (D = sacrificial electron donor).

The kinetics of the catalytic reaction during the first 2–4 h was studied, when using 1, 5, and 18 mg of cluster compound (Figure 5a). With the minimum cluster loading, the hydrogen production is linear, with a production rate of 459 μmol·g^–1^·h^–1^, which approaches the value achieved at 24 h (442 μmol·g^–1^·h^–1^). The linear behavior is maintained when using 5-fold the catalyst amount (Figure S8a) whereas, in this case, the H_2_ production rate is 1 order of magnitude slower. A two-step behavior is observed in the presence of stoichiometric amounts of the Ta_6_ compound (Figure S8b). In this case, the hydrogen production is initially low (<5 μmol·g^–1^), and after 30 min of reaction, the induction period is overcome and the reaction rate increases to a slow reaction rate (24 μmol·g^–1^·h^–1^) in a lineal progression. The easy oxidation of the Ta_6_ cluster to afford 3+ and 4+ oxidized species suggests that oxidative quenching by the electron donor (methanol) would be involved in the three component system. The proposed quenching mechanism (Figure 5b) would comprise a first photoinduced electron transfer reaction step from the cluster excited state to the H^+^, entailing the reduction of the H^+^ and the oxidation of the cluster species in a 3+/4+ comproportionation equilibria (3) and followed by a regeneration step to the 2+ cluster species by the electron donor in a last reaction step.

### Plausible Species Involved in HER

The absorption spectra of hydrated cluster species recorded in pure water and in the presence of acid show the characteristic {Ta_6_Br^i^_12_}^2+^ bands associated with the d–d transitions of the 16 valence electron cluster (VEC) species. These absorption bands correspond to [{Ta_6_Br^i^_12_}(H_2_O)^a^_6_]^2+^ and [{Ta_6_Br^i^_12_}(H_2_O)^a^_5_(OH)^a^]^+^ species present in solution, which are in equilibrium via aqua-hydroxo exchange or an aqua ligand deprotonation reactions (p*K*_a_ = 3.9).^16^ In order to understand their absorption properties, both cluster structures were first optimized by DFT calculations, and we found a good agreement with X-ray data (Table S2). Second, the absorption spectra of the aqua [{Ta_6_Br^i^_12_}(H_2_O)^a^_6_]^2+^ and the hydroxo [{Ta_6_Br^i^_12_}(H_2_O)^a^_5_(OH)^a^]^+^ species were calculated at TD-DFT level (Figures S9a and b), indicating a good match with the experiment in terms of the absorption wavelengths. The intensity ratio (ca. 1.4) between the two NIR bands of the experimental spectra is close to the ratio calculated for the corresponding bands in the calculated spectrum of the [{Ta_6_Br^i^_12_}(H_2_O)^a^_5_(OH)^a^]^+^ species (Figure S9b). This confirms the presence of [{Ta_6_Br^i^_12_}(H_2_O)^a^_6_]^2+^:[{Ta_6_Br^i^_12_}(H_2_O)^a^_5_(OH)^a^]^+^clusters in a 70:30 ratio in solution (Figure S10). This ratio does not even change considering the absorption spectrum in acid conditions (Figure 1a and Figure S4d); therefore, the equilibrium between both [{Ta_6_Br^i^_12_}(H_2_O)^a^_6_]^2+^ and [{Ta_6_Br^i^_12_}(H_2_O)^a^_5_(OH)^a^]^+^ species should be considered in the photoredox transformations in acid conditions. The substitution of a water molecule with a hydroxo group impacts both the geometry and the atomic charge distribution of the cluster. In particular, in [{Ta_6_Br^i^_12_}(H_2_O)^a^_6_]^2+^, the average charge assigned to the Ta atoms, Br atoms, and water molecules is 0.449|e|, −0.162|e|, and 0.209|e|, respectively. Upon apical ligand substitution, the axial Ta atom bonded with the OH group becomes less positive (0.427|e|), and the atom charge of the Ta atom in the *trans* position remains barely the same (0.443|e|), whereas the other four equatorial Ta atoms become more positive in charge (averagely 0.471|e|). Part of the negative charge is distributed over the bridging Br atoms, which become more negatively charged (averagely −0.195|e|), and the water molecules, which become less positively charged (averagely 0.194|e|); the charge attributed to the OH group is −0.382|e|. This inhomogeneity in the charge distribution for the [{Ta_6_Br^i^_12_}(H_2_O)^a^_5_(OH)^a^]^+^ species suggests plausible different catalytic sites within the cluster.

The coordinating ability of methanol can promote the *in situ* generation of methanolic cluster species, since the {Ta_6_Br^i^_12_} cluster core is known to exhibit chemical affinity to alcohol ligands.^12,40,41,47,48^ In the conditions employed for the catalytic experiments, the aqua/methanol ligand exchange reaction was proposed to lead to [{Ta_6_Br^i^_12_}(H_2_O)^a^_*x*_(MeOH)^a^_6–*x*_]^2+^ (*x* = 1 to 5) species at the beginning of the reaction. The absorption spectrum of the [{Ta_6_Br^i^_12_}(H_2_O)^a^_5_(MeOH)]^2+^ species was calculated at the TD-DFT level, showing two absorption NIR bands with similar fingerprints to [{Ta_6_Br^i^_12_}(H_2_O)^a^_6_]^2+^, but with a bathochromic shift of 20–30 nm (Figure S9c). These peaks are not appreciated in the experimental spectra (Figure S5b–e), even registered in the presence of really employed amounts of methanol (4.94 mol·L^–1^) and acid (1.0 mol·L^–1^). Nevertheless, (H_2_O)_5_(MeOH) cluster species could be generated under catalytic conditions, in low concentration, or as transient species in a fast ligand exchange.

Oxidized +3 and +4 clusters are involved in the light-promoted photoredox transformations. To shed further light on the thermodynamics of the proposed ligand exchange reactions in [{Ta_6_Br^i^_12_}(H_2_O)^a^_6_]^*n*+^ (*n* = 2, 3, 4) the changes in enthalpies and Gibbs free energies were calculated (Table 2). The results obtained indicated that aqua-hydroxo exchange is exothermic (entry 1), as expected. The energy balance is also favorable for higher cluster oxidation states (entries 2 and 3). The aqua-methanol ligand exchange reactions were also investigated (entries 4–6). In these cases, the thermochemical data indicate that water/methanol exchange reactions are much less favorable than water/hydroxo exchange reactions. Even if the thermodynamics values for reactions 4 and 6 still favor the formation of methanolic complexes in solution, the formation of methanolic cluster species is less feasible.

**Table 2:** Thermochemical Data (in kJ·mol^–1^) for the Listed Reactions at *T* = 298.15 K and *p* = 1 atm^a^

**entry**	**reaction**	**Δ***H*	**Δ***G*
(1)	[{Ta_6_Br^i^_12_}(H_2_O)^a^_6_]^2+^ + OH^–^ ⇄ [{Ta_6_Br^i^_12_}(H_2_O)^a^_5_(OH)^a^]^+^ + H_2_O	–132.3	–134.1
(2)	[{Ta_6_Br^i^_12_}(H_2_O)^a^_6_]^3+^ + OH^–^ ⇄ [{Ta_6_Br^i^_12_}(H_2_O)^a^_5_(OH)^a^]^2+^ + H_2_O	–171.1	–164.8
(3)	[{Ta_6_Br^i^_12_}(H_2_O)^a^_6_]^4+^ + OH^–^ ⇄ [{Ta_6_Br^i^_12_}(H_2_O)^a^_5_(OH)^a^]^3+^ + H_2_O	–217.6	–222.1
(4)	[{Ta_6_Br^i^_12_}(H_2_O)^a^_6_]^2+^ + MeOH ⇄ [{Ta_6_Br^i^_12_}(H_2_O)^a^_5_(MeOH)^a^]^2+^ + H_2_O	–10.3	- 2.2
(5)	[{Ta_6_Br^i^_12_}(H_2_O)^a^_6_]^3+^ + MeOH ⇄ [{Ta_6_Br^i^_12_}(H_2_O)^a^_5_(MeOH)^a^]^3+^ + H_2_O	–9.4	+9.2
(6)	[{Ta_6_Br^i^_12_}(H_2_O)^a^_6_]^4+^ + MeOH ⇄ [{Ta_6_Br^i^_12_}(H_2_O)^a^_5_(MeOH)^a^]^4+^ + H_2_O	–9.1	–2.3

a Level of theory: DFT M06-2X/Def2-TZVPP (SMD).

The thermochemical parameters of the redox transformations indicate that all the {Ta_6_Br^i^_12_}^2+^/{Ta_6_Br^i^_12_}^3+^ and {Ta_6_Br^i^_12_}^3+^/{Ta_6_Br^i^_12_}^4+^ oxidation reactions, associated with the [{Ta_6_Br^i^_12_}(H_2_O)^a^_6_]^2+^, [{Ta_6_Br^i^_12_}(H_2_O)^a^_5_(OH)^a^]^+^, and [{Ta_6_Br^i^_12_}(H_2_O)^a^_5_(MeOH)^a^]^2+^ precursors, are endothermic (Table S3). This supports the need for an external energy source, such as light, to drive the reaction. It is worth mentioning that the cluster oxidation is ca. 50 kJ·mol^–1^ more favorable when hydroxo-containing clusters are involved, suggesting that these are more energetically accessible intermediates involved in the photoredox reactions studied in this work.

The reaction mechanism proposed for the photoredox transformation was revisited via computational and experimental approaches and involving the aqua-hydroxo Ta_6_Br^i^_12_ cluster core complexes, as the most plausible species involved in the catalytic transformation.^13^ In a first step, upon the photoexcitation of the {Ta_6_Br^i^_12_}^2+^ cluster species (5), a deactivation (6) may follow competing with a two-electron oxidation of {Ta_6_Br^i^_12_}^2+^ into {Ta_6_Br^i^_12_}^4+^ (eq 7). Photoluminescence measurements of the catalytic reaction mixture were registered and showed no cluster emission within the visible and near-infrared (NIR) window (Figure S11), which confirms a fast photoinduced electron transfer process.
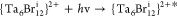
5
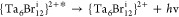
6
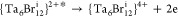
7

Subsequent reaction steps of the mechanism proposed for the hydrogen production reaction and the corresponding reaction energies are depicted in Table 3. First, the two-electron oxidation of the {Ta_6_Br^i^_12_}^2+^ hydroxo cluster to a water molecule was proposed as a key step to lead the formation of H^–^ and both *trans-* and *cis*-[{Ta_6_Br^i^_12_}(H_2_O)^a^_4_(OH)^a^_2_]^2+^ isomers (Table 3, eqs 8 and 10, respectively).^13^ Both reactions are highly thermodynamically favored, with small differences in enthalpy and Gibbs free energy between them (5.3 and 1.5 kJ·mol^–1^, respectively). The formation of hydrido Ta_6_ species has not been reported until present and was investigated in the gas phase by ESI-MS from a solution composed by [{Ta_6_Br^i^_12_}Br^a^_2_(H_2_O)^a^_4_]·4H_2_O and methanol (20% v/v). One peak was detected at *m*/*z* = 2205.8347 Da in the mass spectrum, and it was associated with [{Ta_6_Br^i^_12_}(MeOH)_5_(H)]^+^ (Figure S12). The coordinative ability of hydrides could be ascribed to the ESI conditions, and the generation of plausible hydrido cluster intermediates under irradiation conditions is unknown. The reduction of a water molecule can be accomplished by hydride generation promoted by this two-electron cluster transfer. The two protons from water molecules are involved, with concomitant generation of H_2_ and 2 OH^–^ in solution. Thus, the second reaction (eqs 9 and 11) involves protonation of the dihydroxo intermediates to give [{Ta_6_Br^i^_12_}(H_2_O)^a^_5_(OH)^a^]^3+^. The species generated in the two-step oxidation–protonation reactions contribute to the production of aqua-hydroxo {Ta_6_Br^i^_12_}^3+^ cluster species through a comproportionation process (eq 12), as proposed in 3. The formation of the species listed in Table 3 explains the slow reaction rate and the induction period described from the kinetic study of the stoichiometric reaction (Figure S8b). The {Ta_6_Br^i^_12_}^3+^ cluster species formed would react with the electron donor (MeOH) sacrificial agent, present in excess in the reaction mixture, to recover the initial cluster within the catalytic cycle, in agreement with the oxidative quenching mechanism proposed in Figure 5b.

**Table 3:** Thermochemical Data (in kJ·mol^–1^) for the Listed Reactions at *T* = 298.15 K and *p* = 1 atm^a^

**eq**	**reaction**	**Δ***H*	**Δ***G*
(8)	[{Ta_6_Br^i^_12_}(H_2_O)^a^_5_(OH)^a^]^+^ ⇄ *trans*-[{Ta_6_Br^i^_12_}(H_2_O)^a^_4_(OH)^a^_2_]^2+^ + H^–^	–927.0	–961.6
(9)	*trans*-[{Ta_6_Br^i^_12_}(H_2_O)^a^_4_(OH)^a^_2_]^2+^ + H_3_O^+^ ⇄ [{Ta_6_Br^i^_12_}(H_2_O)^a^_5_(OH)^a^]^3+^ + H_2_O	–45.6	–41.2
(10)	[{Ta_6_Br^i^_12_}(H_2_O)^a^_5_(OH)^a^]^+^ ⇄ *cis*-[{Ta_6_Br^i^_12_}(H_2_O)^a^_4_(OH)^a^_2_]^2+^ + H^–^	–921.8	–960.1
(11)	*cis*-[{Ta_6_Br^i^_12_}(H_2_O)^a^_4_(OH)^a^_2_]^2+^ + H_3_O^+^ ⇄ [{Ta_6_Br^i^_12_}(H_2_O)^a^_5_(OH)^a^]^3+^ + H_2_O	–50.9	–42.7
(12)	[{Ta_6_Br^i^_12_}(H_2_O)^a^_5_(OH)^a^]^+^ + [{Ta_6_Br^i^_12_}(H_2_O)^a^_5_(OH)^a^]^3+^ ⇄ 2 [{Ta_6_Br^i^_12_}(H_2_O)^a^_5_(OH)^a^]^2+^	–56.2	–65.2

a Level of theory: DFT M06-2X/Def2-TZVPP (SMD).

## Conclusions

The performance of [{Ta_6_Br^i^_12_}Br^a^_2_(H_2_O)^a^_4_]·4H_2_O toward photoassisted hydrogen generation was screened in aqueous media by using methanol, acetic acid, and lactic acid as sacrificial electron donors, with and without the assistance of acids, such as HBr and phosphoric acid. The use of MeOH/HBr mixtures was proposed as optimal since the largest H_2_ production was achieved, and the robustness of the tantalum cluster species involved was maintained after the catalytic reaction. *In situ/in operando* reactivity studies of [{Ta_6_Br^i^_12_}Br^a^_2_(H_2_O)^a^_4_]·4H_2_O with hydrobromic acid confirmed that the photoredox reaction involved in the HER takes place in the presence of HBr, and the increase of the acid concentration favors cluster precipitation with a concomitant decrease in the hydrogen production. The optimization of the amounts of acid and methanol used in the HER process was identified via RSM to improve the yield in the H_2_ production. The models showed a good predictability with a high correlation between the experimental and theoretical values at a 95% confidence level. Optimal MeOH and HBr concentrations are 4.83 and 0.7 mol·L^–1^, respectively. Optimal H_2_ production was achieved using catalytic amounts of the Ta_6_ material (442 μmol·g^–1^·h^–1^, TOF of 3 × 10^–4^ s^–1^). The catalytic efficiency of the brominated Ta_6_ cluster compound in homogeneous conditions improves in 1 order of magnitude the activities described for other Ta-based solids assisted by noble metals in heterogeneous conditions, and up to 126-fold with that obtained from the supported cluster catalyst ({Ta_6_Br^i^_12_}@GO) with the presence of water vapor. To the best of our knowledge, this is the highest production achieved by tantalum-based materials. Aqua-hydroxo cluster intermediates were proposed as the most plausible active species involved in the photoredox mechanism on the basis of computational and experimental results. This mechanistic study highlights the importance of the surrounding ligands of the Ta_6_Br^i^_12_ cluster cores, and opens new possibilities to enhance the catalytic efficiency of the tantalum cluster sites by modifying the cluster chemical composition. In summary, the optimal photocatalytic performance for the homogeneous Ta_6_Br^i^_12_ system for the HER reaction from aqueous protons was determined by experimental and computational techniques, demonstrating the stability of the catalytic system. The results obtained represents a step ahead with respect to Vogler’s photoredox studies and shed light on the knowledge and understanding of the reactivity of octahedral Ta clusters with halides.

## Data Availability

Not applicable.

## Supporting Information Available

Experimental setup, control experiments, CV, absorption and ESI-MS spectra, RSM data, H_2_ production rates, computational data, photoluminescence spectra and references (PDF)
