# Supplementary material for: Octahedral Tantalum Bromide Clusters as Catalysts for Light-Driven Hydrogen Evolution
Source: Inorg Chem. 2023 Nov 7;62(46):19060–9. doi: 10.1021/acs.inorgchem.3c03045 (PMC10664069; doi:10.1021/acs.inorgchem.3c03045)
Supplement: Supplementary file 1 — ic3c03045_si_001.pdf [file ic3c03045_si_001.pdf]

Supporting Information

Octahedral Tantalum Bromide Clusters as Catalysts for Light-Driven Hydrogen Evolution

Jhon Sebastián Hernández[1], Daniela Guevara[1], Maxim Shamshurin[2], Enrico Benassi[3,*], Maxim N. Sokolov[2], and Marta Feliz[1,*]

[1] Instituto de Tecnología Química, Universitat Politècnica de València - Consejo Superior de Investigaciones Científicas (UPV-CSIC), Avd. de los Naranjos s/n, 46022 Valencia, Spain
[2] Nikolaev Institute of Inorganic Chemistry SB RAS, 3 Akad. Lavrentiev Ave., 630090 Novosibirsk, Russian Federation.
[3] Novosibirsk State University, 2 Pirogov Str., 630090 Novosibirsk, Russian Federation

* Corresponding authors:
Enrico Benassi: ebenassi3@gmail.com
Marta Feliz: mfeliz@itq.upv.es

1. Figures and Tables.

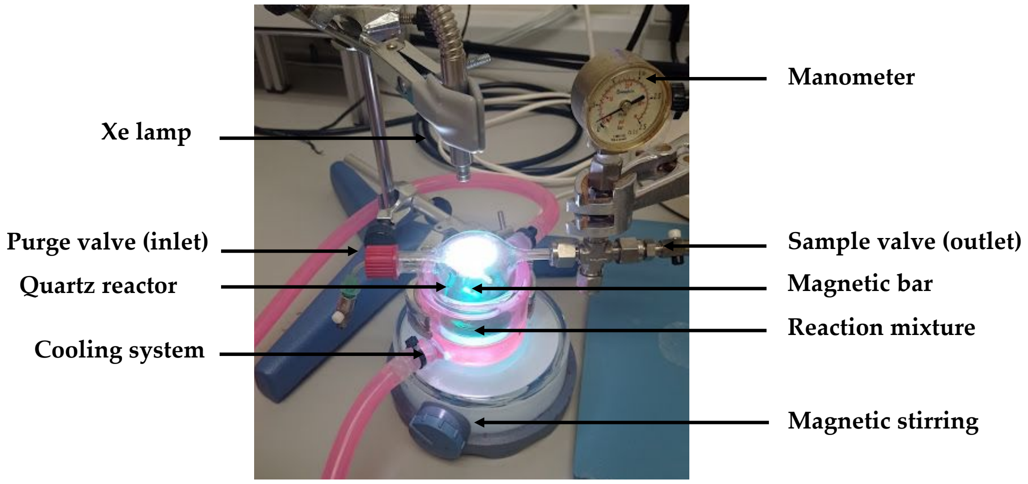

Figure S1. Experimental setup for the photocatalytic hydrogen production performed in homogeneous conditions.

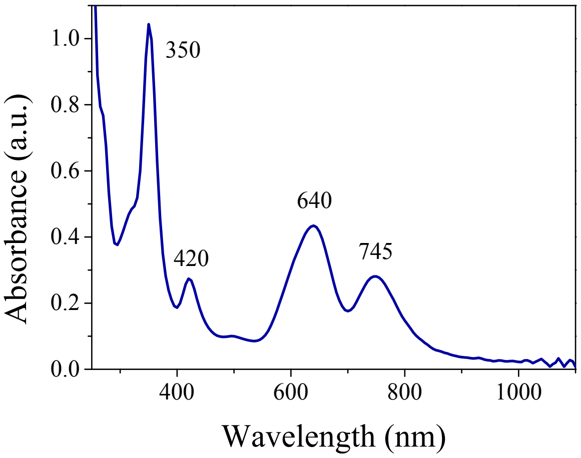

Figure S2. Absorption spectrum of the aqueous $\{Ta_6Br^i{}_{12}\}^{2+}$ cluster complex in water (1.2×10^{-4} M)

Table S1. Amount of H_2 produced in control tests done under standard photocatalytic conditions.

Test	Control conditions				H_2 produced (μmol)
	Photocatalyst $\{Ta_6Br^i_{12}\}$	Light	MeOH	HBr	
1	No	Yes	Yes	Yes	0.06
2	Yes	No	Yes	Yes	0.10
3	Yes	Yes	No	Yes	0.06
4	Yes	No	No	No	0.59
5	No	Yes	No	No	0.04
6	Yes	No	Yes	No	1.83
7	Yes	No	No	Yes	ND [a]
8	No	No	Yes	Yes	ND [a]

[a] Not detected

Table S2. Coefficients of regression model and their significance.[a]

	Estimate	Std. Error	t value	Pr(>ltl)	
Intercept	-92.0481	80.8434	-1.13860	0.298282	.
Bloque2	33.9346	21.01630	1.61470	0.1575083	
MeOH	166.8089	20.44550	8.15870	0.0001824	***
HBr	402.3458	83.38990	4.82490	0.0029255	**
MeOH:HBr	-3.6721	10.02450	-0.36630	0.7267041	
MeOH^2	-16.9964	1.7686	-9.60990	7.26E-05	***
HBr^2	-285.3643	32.66320	-8.73660	0.0001244	***

[a] Multiple R-squared: 0.982, Adjusted R-squared: 0.964 F-statistic: 54.58 on 6 and 6 DF, p-value: 5.668e-05

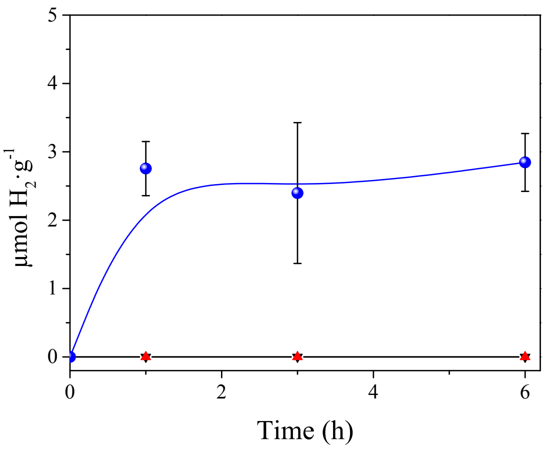

Figure S3. Amount of H_2 evolved under irradiation conditions for $[\{Ta_6Br^i_{12}\}Br^a_2(H_2O)^a_4]$ (in black), $[\{Ta_6Br^i_{12}\}Br^a_2(H_2O)^a_4]$/HBr (in blue) and water (in red).

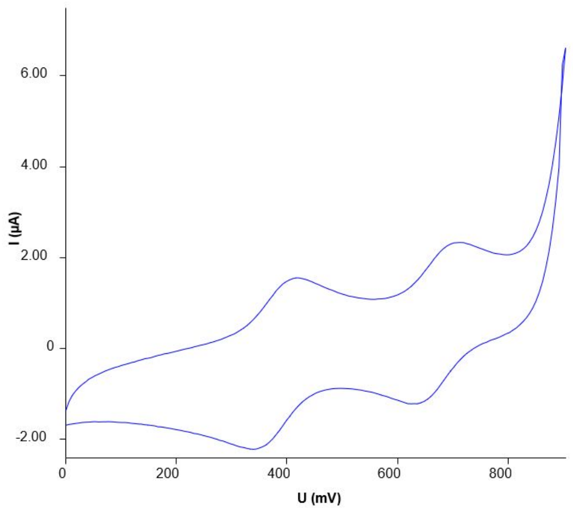

Figure S4. CV of $[\{Ta_6Br_{12}\}(H_2O)_6]^{2+}$ in water registered in the range from 0 to 0.90 V and at potential scan rate of 100 mV/s.

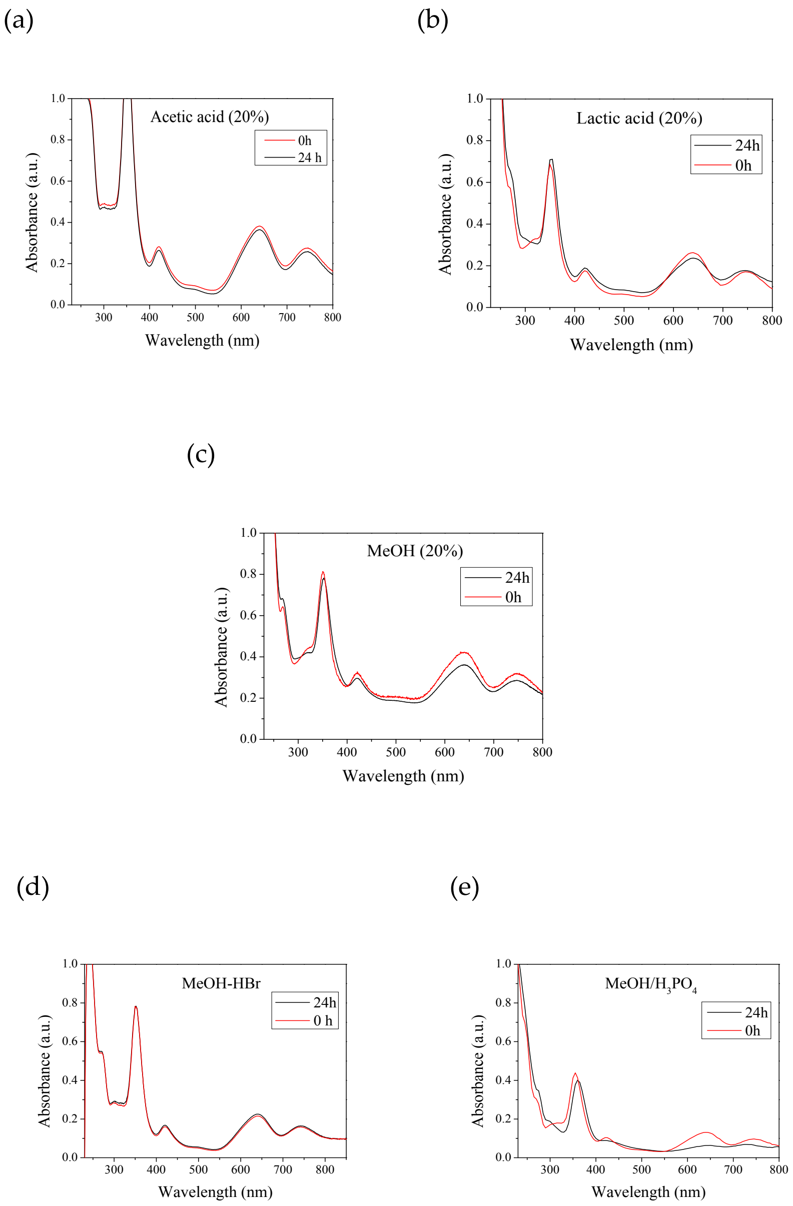

Figure S5. UV-Vis spectra of $[\{Ta_6Br^i_{12}\}Br^a_2(H_2O)^a_4]$ in presence of (a) acetic acid (3.50 mol·L^{-1}), (b) lactic acid (2.66 mol·L^{-1}), (c) methanol (4.94 mol·L^{-1}) (d) methanol (4.94 mol·L^{-1})/HBr (1.0 mol·L^{-1}) and (e) methanol (4.94 mol·L^{-1})/H_3PO_4 (3.84 mol·L^{-1}).

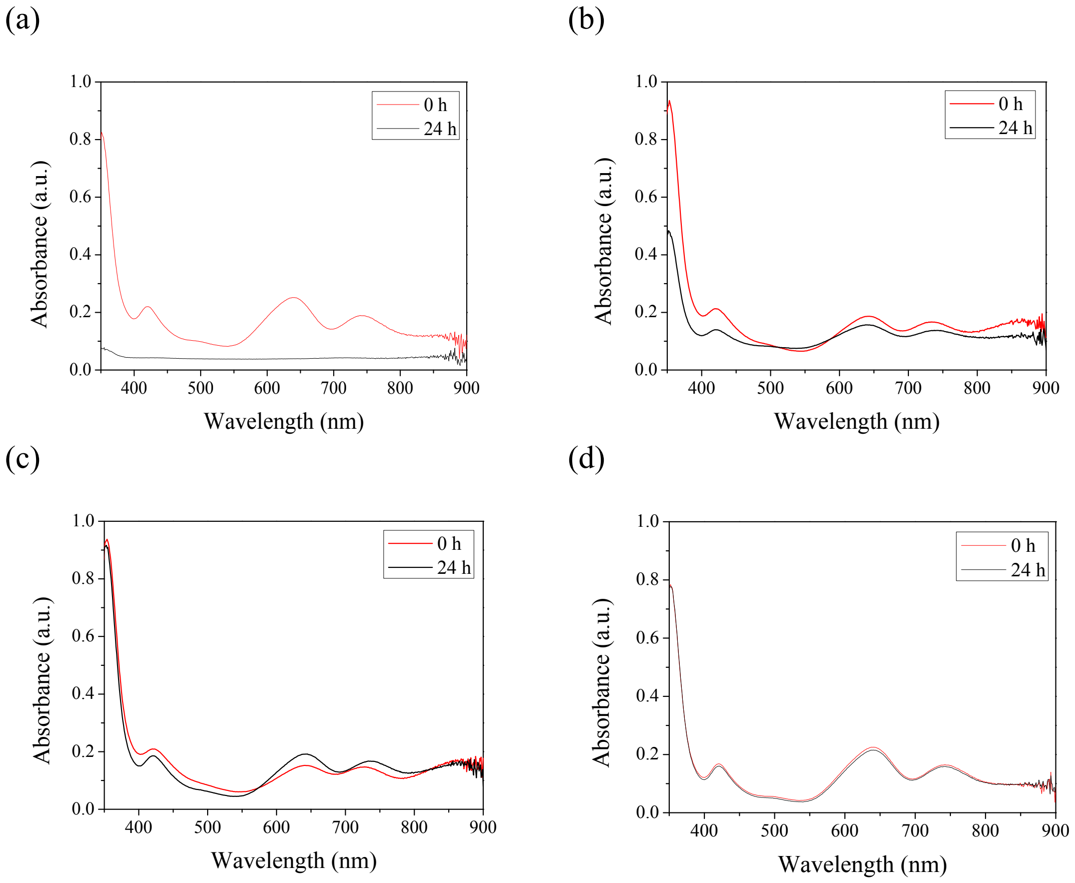

Figure S6. UV-Vis spectra of $[\{Ta_6Br^i_{12}\}Br^a_2(H_2O)^a_4]$ in methanol/HBr mixtures: (a) 4.94 mol·L^{-1}/1.9 mol·L^{-1}, (b) 7.41 mol·L^{-1}/1.5 mol·L^{-1}, and (c) 8.65 mol·L^{-1}/1.0 mol·L^{-1}; (d) 4.83 mol·L^{-1} and 0.7 mol·L^{-1}.

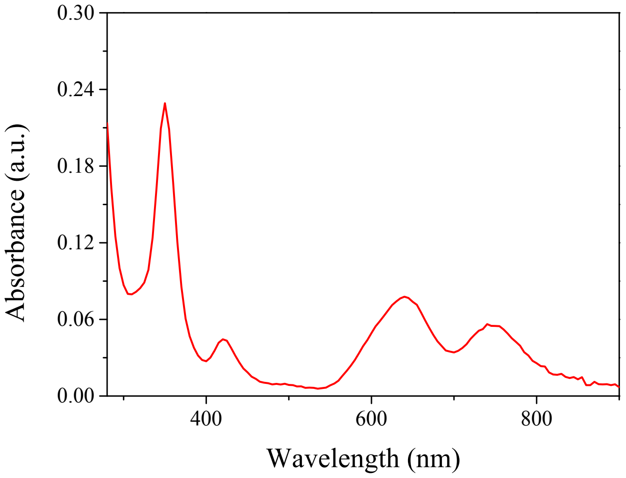

Figure S7. UV-Vis spectrum of $[\{Ta_6Br^i_{12}\}Br^a_2(H_2O)^a_4]$ after 24 h reaction under optimized catalytic conditions.

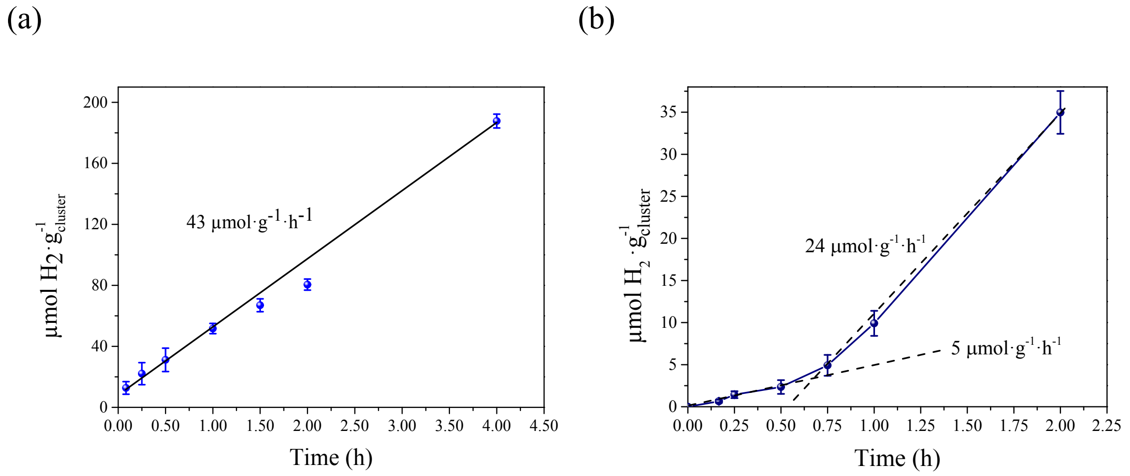

Figure S8. Kinetic study and reaction rates of the UV-Vis light driven H_2 generation in the presence of (a) 5 mg and (b) 18 mg of $[(Ta_6Br^i_{12}\}Br^a_2(H_2O)^a_4]\cdot 4H_2O$.

Table S3. Average interatomic distances in $[\{Ta_6Br_{12}\}(H_2O)_6]^{2+}$ clusters (in 10^{-10} m units). Comparison between reference experimental values from the literature and values computed in this work.

	**ref.**	**Ta-Ta**	**Ta-Br**	**Ta-O**
exp.	(1)	2.903 ± 0.008	2.607 ± 0.008	2.246 ± 0.023
	(2)	2.893 ± 0.006	2.601 ± 0.006	2.285 ± 0.029
	(3)	2.903 ± 0.014	2.607 ± 0.005	2.294 ± 0.045
comp.	this work	2.913 ± 0.001	2.607 ± 0.003	2.290 ± 0.004

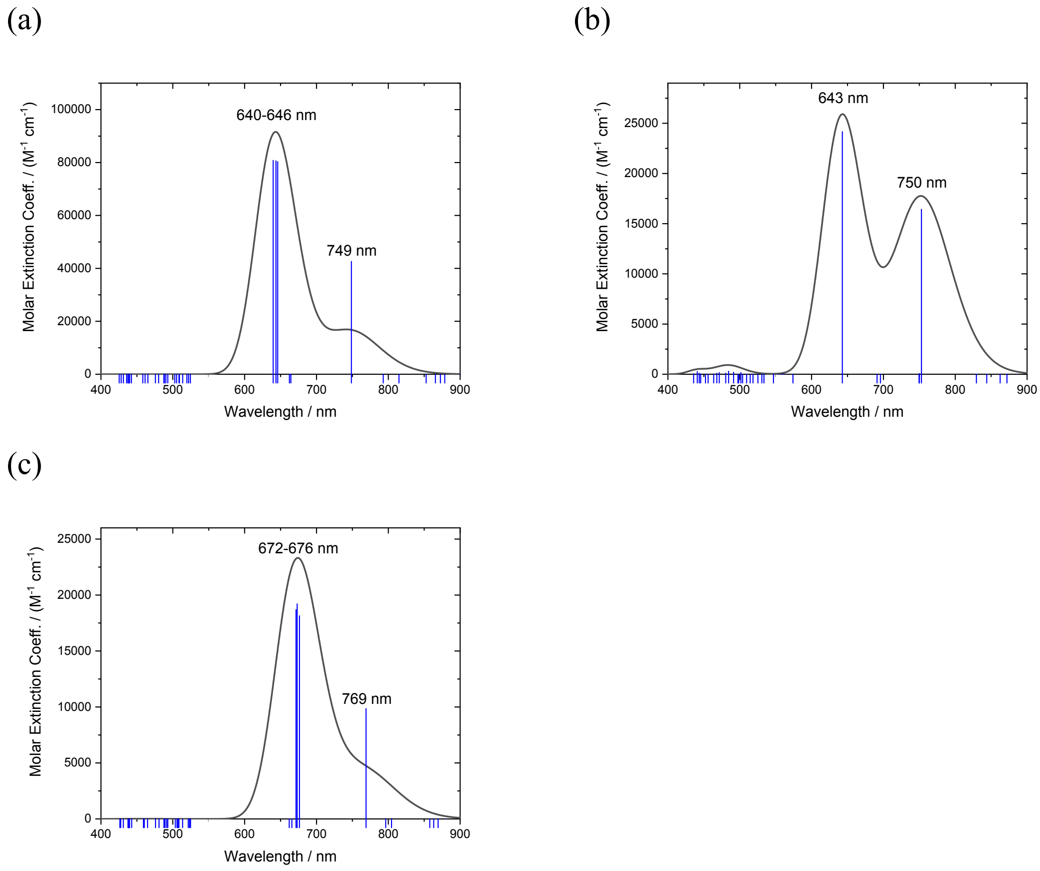

Figure S9. Computed (TD-DFT) absorption spectra for (a) $[\{Ta_6Br^i_{12}\}(H_2O)^a_6]^{2+}$, (b) $[\{Ta_6Br^i_{12}\}(H_2O)^a_5(OH)^a]^{+}$ and (c) $[\{Ta_6Br^i_{12}\}(H_2O)^a_5(MeOH)^a]^{2+}$ species.

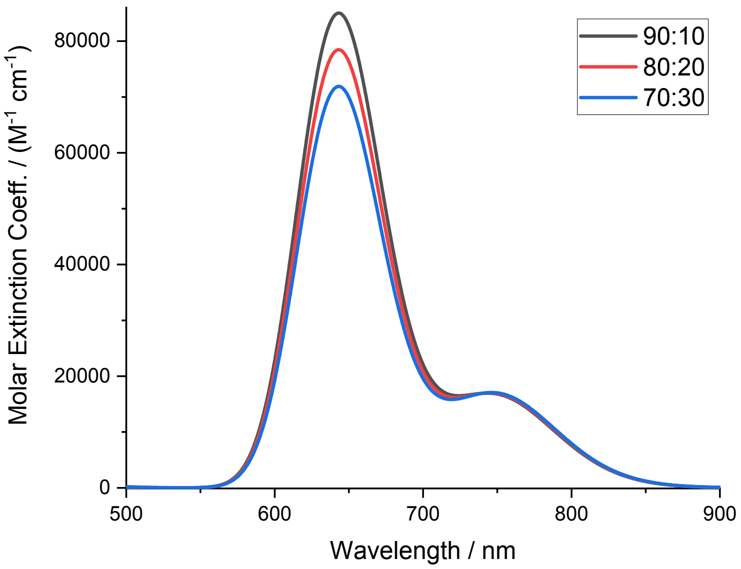

Figure S10. TD-DFT simulation of the spectra of the mixture of Ta bromide clusters at different molar concentrations.

Table S4. Thermochemical data ($kJ \cdot mol^{-1}$) computed for the listed reactions at $T = 298.15$ K and $p = 1$ atm.

Entry	Reaction	ΔH	ΔG
(1)	$[\{Ta_6Br^i_{12}\}(H_2O)^a_6]^{+2} \rightleftarrows [\{Ta_6Br^i_{12}\}(H_2O)^a_6]^{+3} + e^-$	514.3	506.0
(2)	$[\{Ta_6Br^i_{12}\}(H_2O)^a_6]^{+3} \rightleftarrows [\{Ta_6Br^i_{12}\}(H_2O)^a_6]^{+4} + e^-$	578.3	597.8
(3)	$[\{Ta_6Br^i_{12}\}(H_2O)^a_5(OH)^a]^{+1} \rightleftarrows [\{Ta_6Br^i_{12}\}(H_2O)^a_5(OH)^a]^{+2} + e^-$	475.6	475.3
(4)	$[\{Ta_6Br^i_{12}\}(H_2O)^a_5(OH)^a]^{+2} \rightleftarrows [\{Ta_6Br^i_{12}\}(H_2O)^a_5(OH)^a]^{+3} + e^-$	531.8	540.5
(5)	$[\{Ta_6Br^i_{12}\}(H_2O)^a_5(MeOH)^a]^{+2} \rightleftarrows [\{Ta_6Br^i_{12}\}(H_2O)^a_5(MeOH)^a]^{+3} + e^-$	515.2	517.3
(6)	$[\{Ta_6Br^i_{12}\}(H_2O)^a_5(MeOH)^a]^{+3} \rightleftarrows [\{Ta_6Br^i_{12}\}(H_2O)^a_5(MeOH)^a]^{+4} + e^-$	578.6	586.3

(a)

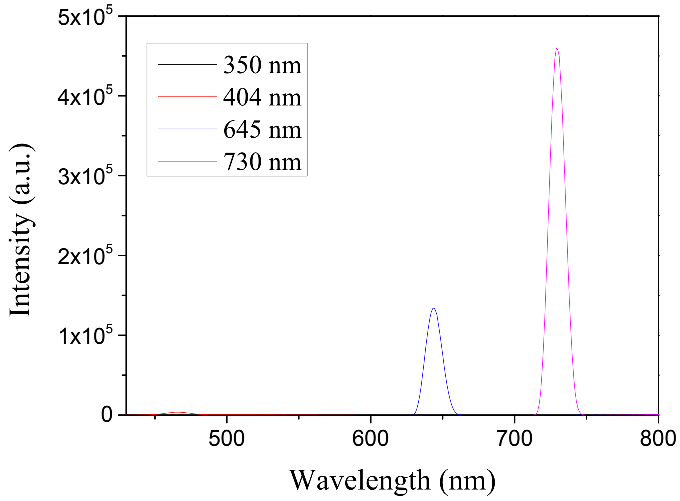

(b)

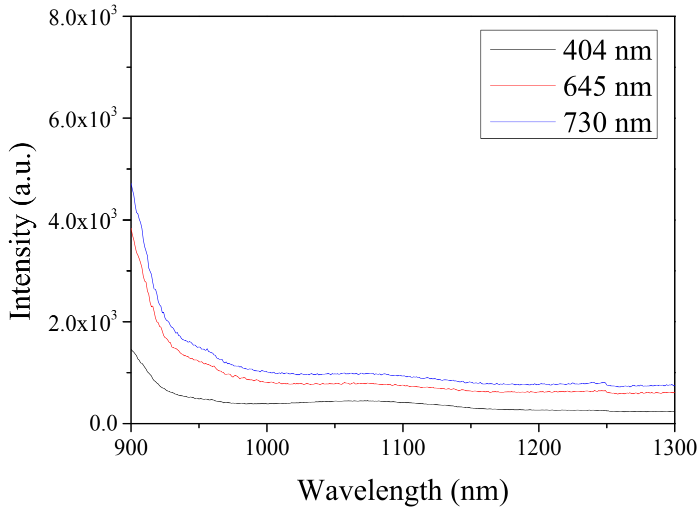

Figure S11. Photoluminescence spectra of optimized reaction MeOH/HBr mixture registered at different excitation wavelengths and under Ar with a (a) visible detector and (b) InGaAs detector.

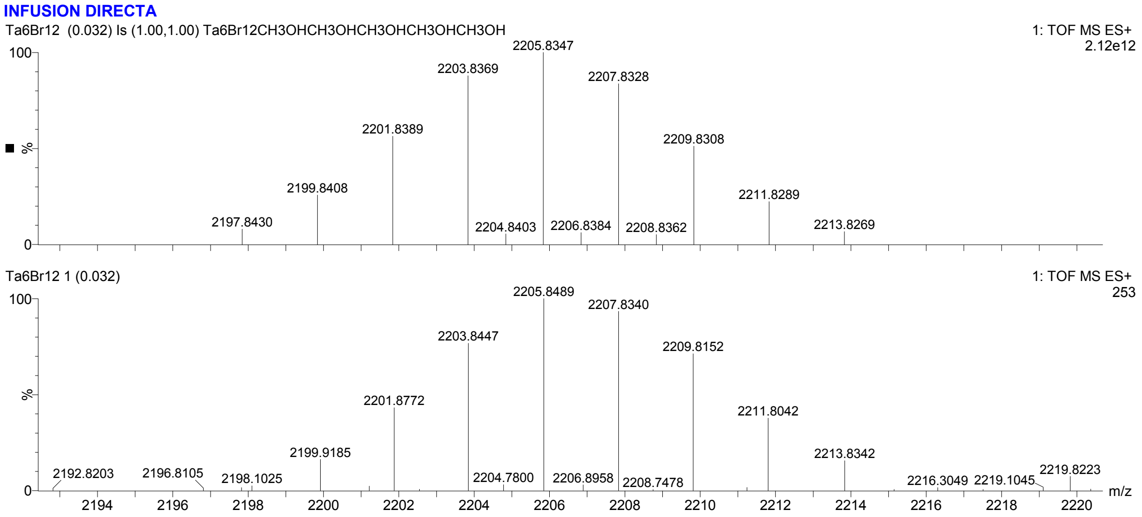

Figure S12. Experimental (bottom) and simulated (top) mass spectra of the peak at *m/z* = 2205.85 uma.

2. References

1. Vojnovic M, Jozic D, Giester G, Peric B, Planinic P, Brnicevic N. Bis(tetramethylammonium) hexaaquadodeca-[mu]-bromo-octahedro-hexatantalum tetrabromide dihydrate. Acta Crystallographica Section C. 2002;58(4):m219-m20.
2. Anyushin AV, Sokolov MN, Peresypkina EV, Fedin VP. Crystal structure of a tantal cluster with tetraphenylborate-anion: $[Ta_6Br_{12}(H_2O)_6](BPh_4)_2{\cdot}4H_2O$. Journal of Structural Chemistry. 2013;54(2):454-8.
3. Moussawi MA, Leclerc-Laronze N, Floquet S, Abramov PA, Sokolov MN, Cordier S, Ponchel A, Monflier E, Bricout H, Landy D, Haouas M, Marrot J, Cadot E. Polyoxometalate, Cationic Cluster, and γ-Cyclodextrin: From Primary Interactions to Supramolecular Hybrid Materials. Journal of the American Chemical Society. 2017;139(36):12793-803.
